# Metagenomic 16S rDNA amplicon data on bacterial diversity profiling and its predicted metabolic functions of varillales in Allpahuayo-Mishana National Reserve

**DOI:** 10.1016/j.dib.2020.105625

**Published:** 2020-04-28

**Authors:** Juan C. Castro, J. Dylan Maddox, Hicler N. Rodríguez, Richard B. Orbe, Gad E. Grandez, Kevin A. Feldheim, Marianela Cobos, Jae D. Paredes, Carlos G. Castro, Jorge L. Marapara, Pedro M. Adrianzén, Janeth Braga

**Affiliations:** a Unidad Especializada de Biotecnología, Centro de Investigación de Recursos Naturales de la Amazonía (CIRNA), Universidad Nacional de la Amazonia Peruana (UNAP), Iquitos, Perú; b Laboratorio de Biotecnología y Bioenergética (LBB), Universidad Científica del Perú (UCP), Iquitos, Perú; c Pritzker Laboratory for Molecular Systematics and Evolution, Field Museum of Natural History, 1400 S. Lake Shore Drive, Chicago, IL 60605, USA; d Environmental Sciences, American Public University System, Charles Town, WV 25414, USA

**Keywords:** Metagenomics, 16S rRNA, Peruvian amazon, Soil microbiome, Tropical forest, Varillales, White-sand forests

## Abstract

The white-sands forests or varillales of the Peruvian Amazon are characterized by their distinct physical characteristics, patchy distribution, and endemism [1, 2]. Much research has been conducted on the specialized plant and animal communities that inhabit these ecosystems, yet their soil microbiomes have yet to be studied. Here we provide metagenomic 16S rDNA amplicon data of soil microbiomes from three types of varillales in Allpahuayo-Mishana National Reserve near Iquitos, Peru. Composite soil samples were collected from very low varillal, high-dry varillal, and high-wet varillal. Purified metagenomic DNA was used to prepare and sequence 16S rDNA metagenomic libraries on the Illumina MiqSeq platform. Raw paired-endsequences were analyzed using the Metagenomics RAST server (MG-RAST) and Parallel-Meta3 software and revealed the existence of a high percentage of undiscovered sequences, potentially indicating specialized bacterial communities in these forests. Also, were predicted several metabolic functions in this dataset. The raw sequence data in fastq format is available in the public repository Discover Mendeley Data (https://data.mendeley.com/datasets/syktzxcnp6/2). Also, is available at NCBI's Sequence Read Archive (SRA) with accession numbers SRX7891206 (very low varillal), SRX7891207 (high-dry varillal), and SRX7891208 (high-wet varillal).

Specifications Table

**Table:** 

Subject	Genetics, Genomics and Molecular Biology
Specific subject area	Soil Metagenomics
Type of data	Figures and 16S rDNA amplicon sequencing data
How data were acquired	Soil samples were collected from three varillal forest types of Allpahuayo-Mishana National Reserve. The metagenomic DNA was isolated using standardized protocols, and sequenced on Illumina Miseq platform
Data format	Raw data in fastq format were deposited in the public repository Discover Mendeley Data (https://data.mendeley.com/datasets/syktzxcnp6/2). Also, raw data is available in NCBI (https://www.ncbi.nlm.nih.gov/Traces/study/?acc=PRJNA611870&o=acc_s%3Aa)
Parameters for data collection	Metagenomic DNA isolated from soil samples were prepared by amplifying the V3–V4 region of the 16S rDNA gene paired-end sequenced on an Illumina MiSeq platform.
Description of data collection	Filtered sequence reads were analysed using bioinformatics tools (i.e., MG-RAST analysis, Parallel-Meta3 software) of the NGS data.
Data source location	Institution: Universidad Nacional de la Amazonia Peruana City/Town/Region: Iquitos/Maynas/Loreto Region Country: Peru Latitude and longitude (and GPS coordinates) for collected samples/data: 1. very low varillal (3°57′54.293"S, 73°26′10.110"W) 2. high-dry varillal (3°58′33.185"S, 73°25′37.165"W) 3. high-wet varillal (3°58′21.535"S, 73°25′54.369"W)
Data accessibility	Raw sequencing data are hosted in the public repository Discover Mendeley Data with direct URL to data: https://data.mendeley.com/datasets/syktzxcnp6/2 Also, raw sequencing data is available at NCBI under the BioProject No. PRJNA611870 (https://www.ncbi.nlm.nih.gov/Traces/study/?acc=PRJNA611870&o=acc_s%3Aa). SRA accession numbers: SRX7891206 (very low varillal): https://www.ncbi.nlm.nih.gov/sra/SRX7891206 SRX7891207 (high-dry varillal): https://www.ncbi.nlm.nih.gov/sra/SRX7891207 SRX7891208 (high-wet varillal): https://www.ncbi.nlm.nih.gov/sra/SRX7891208

## Value of the data

• This is the first metagenomic 16S rDNA amplicon data on bacterial profiling and its predicted metabolic functions of varillales in Allpahuayo-Mishana National Reserve of the Peruvian Amazon.
• These data provide valuable information on the bacterial diversity and their metabolic functions of varillales in Allpahuayo-Mishana National Reserve of the Peruvian Amazon.
• Metagenomic 16S rDNA amplicon data revealed a high percentage of undiscovered sequences which may indicate varillales contain specialized bacterial communities.

## 1 Data Description

**Fig. 1:**
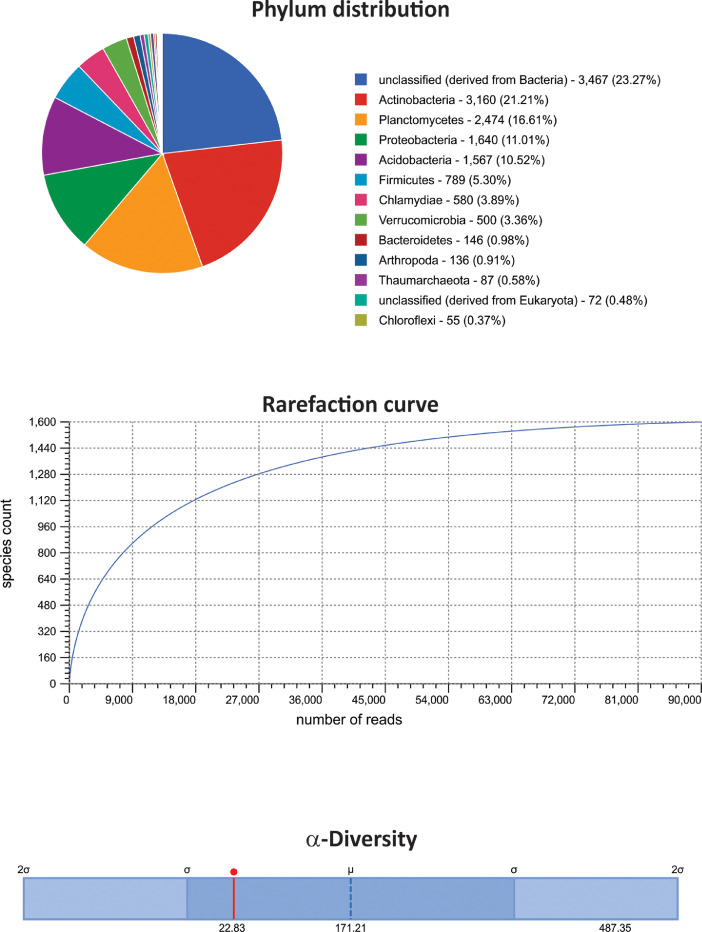
Phylum levels, rarefaction curves and α-diversity of a very low varillal in Allpahuayo-Mishana National Reserve.

**Fig. 2:**
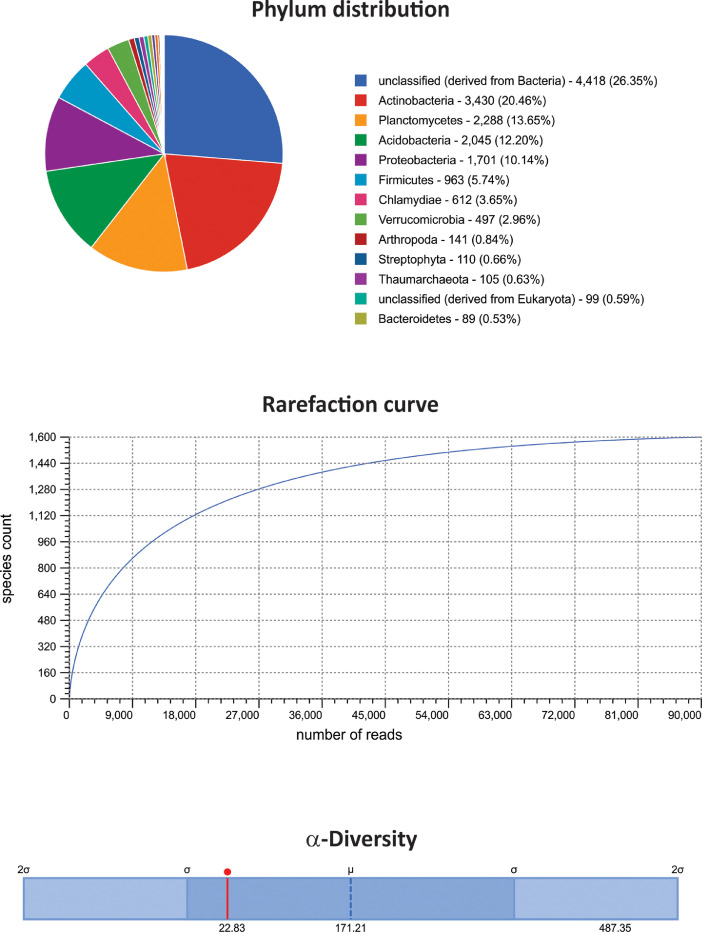
Phylum levels, rarefaction curves and α-diversity of a high-dry varillal in Allpahuayo-Mishana National Reserve.

**Fig. 3:**
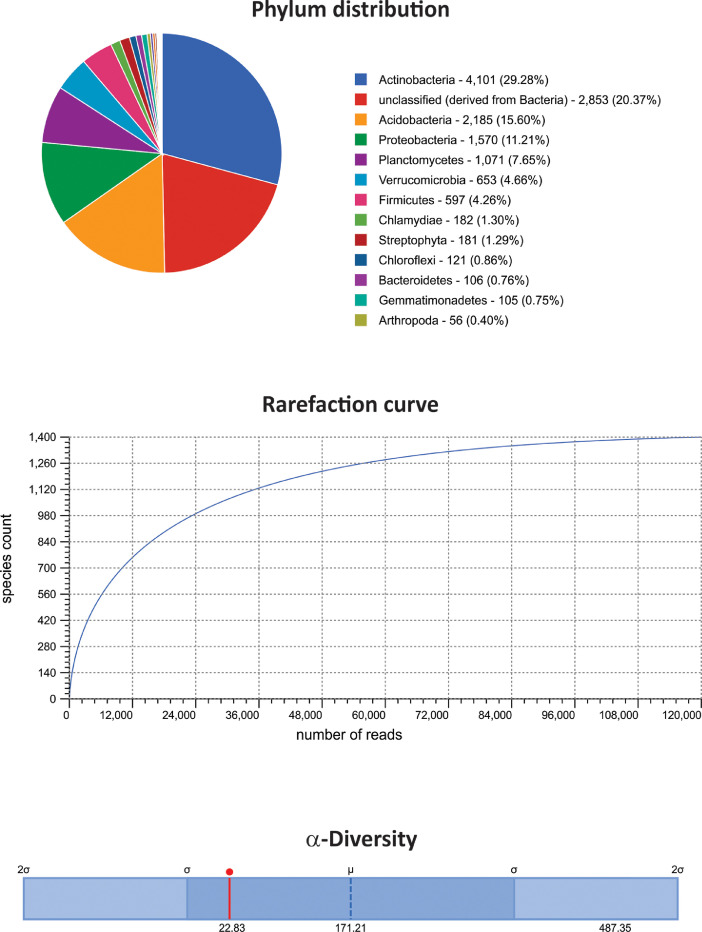
Phylum levels, rarefaction curves and α-diversity of a high-wet varillal in Allpahuayo-Mishana National Reserve.

**Fig. 4:**
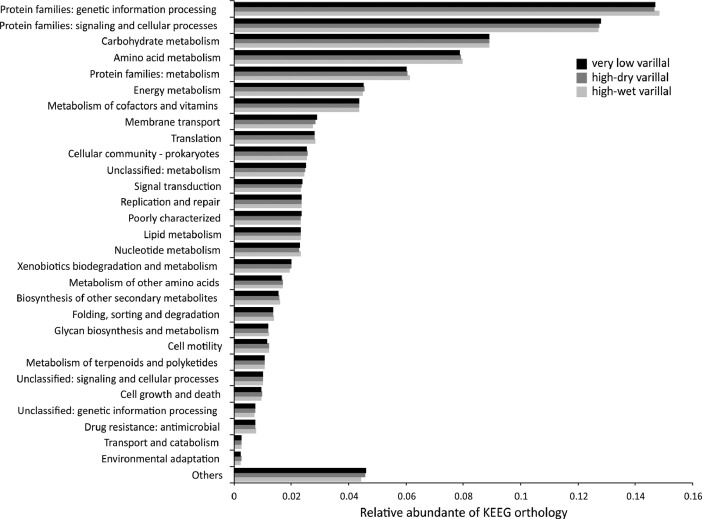
Predicted metabolic functions from three types of varillales in Allpahuayo-Mishana National Reserve.

The dataset contains raw paired-end sequencing data acquired through the V3–V4 region of the 16S rDNA gene of metagenomic DNA isolated from three type of white-sand forests or varillales. The raw sequencing data contain 297,864 sequences totalling 5,966,319 base pairs with an average length of 200 bp. The data files (reads in FASTQ format) were deposited at the public repository Discover Mendeley Data (https://data.mendeley.com/datasets/syktzxcnp6/2) and the NCBI database (https://www.ncbi.nlm.nih.gov/Traces/study/?acc=PRJNA611870&o=acc_s%3Aa) under the BioProject No. PRJNA611870, BioSample accession numbers: SAMN14351537, SAMN14351538, and SAMN14351539; and SRA accession numbers: SRX7891206 (very low varillal), SRX7891207 (high-dry varillal), and SRX7891208 (high-wet varillal). MG-RAST analysis showed that a considerable proportion of sequences were unknown (≈20%). Among the identified sequences, Bacteria (98.4%) and Archaea (0.26%) comprised the majority of the representative kingdoms. The dataset includes data at phylum levels, rarefaction curves and α-diversity results from the very low varillal (Fig. 1), high-dry varillal (Fig. 2), and high-wet varillal (Fig. 3). Additionally, in this dataset were predicted several metabolic functions, such as genetic information processing, carbohydrate metabolism, energy metabolism, etc. (Fig. 4).

## 2 Experimental Design, Materials, and Methods

### 2.1 Sample collection

In this dataset, soil samples were collected from varialles of Allpahuayo-Mishana National Reserve (Supplementary Fig. S1), which is located in a lowland tropical rain forest of the Peruvian Amazon between 130 and 153 m.a.s.l. Soil samples were obtained from three types of varialles as classified by [1]: 1) very low varillal (3°57′54.293``S, 73°26′10.110''W), which is characterized by a high density of small forest trees (height < 5 m) and an organic soil horizon thickness >11 cm ; 2) high-dry varillal (3°58′33.185``S, 73°25′37.165''W), which is characterized by larger forest trees (height >15 m) and an organic soil horizon thickness ≤11 cm; and 3) high-wet varillal (3°58′21.535``S, 73°25′54.369''W), which is also characterized by larger forest trees (height >15 m) but is differentiated by an organic soil horizon thickness >11 cm. Samples were obtained in October 2018 during the high water level season. In order to obtain a representative sample of soil bacterial diversity, thirteen soil cores (10 cm in diameter and 10 cm in depth) were collected in each varillal. The first soil core was designated the reference point for geographic coordinates. The remaining soil cores were sampled at five meter intervals in each cardinal direction with three soil cores obtained in each direction. All thirteen samples from a given reference point were pooled together, homogenized into a composite soil sample per varillal forest type and then passed through a 2 mm meshed sieve (Supplementary Fig. S2). The meshed soil samples were preserved temporarily at −20°C for further studies.

### 2.2 Metagenomic DNA isolation

Metagenomic DNA was isolated from composite soil samples following the protocol of Devi et al., [3]. In addition, to remove humic and fulvic acids contamination and exclude smaller fragments, partially purified metagenomic DNA was subjected to agarose gel (0.6%) electrophoresis for 30 min at 100 V and DNA fragments >20,000 bp were cut away using a sterile scalpel, placed in 2 mL microtubes, and purified with PureLink™ Quick Gel Extraction Kit (Invitrogen™, Catalog: K210012) following the manufacturer's instructions. Quality and quantity of the purified metagenomic DNA (size approximately to 10,000 bp) were verified by both electrophoretic and spectrophotometric analysis using a NanoDrop 2000 (Thermo Scientific).

### 2.3 Library preparation and next-generation DNA sequencing

Amplicon libraries were prepared following the 16S Metagenomics Sequencing Library preparation protocol (Part # 15044223 B). First, metagenomic DNA was amplified using primers designed to target 16S rDNA V3 and V4 regions [4]: 16S rDNA Amplicon PCR Forward Primer = 5′-TCGTCGGCAGCGTCAGATGTGTATAAGAGACAGCCTACGGGNGGCWGCAG-3′, 16S rDNA Amplicon PCR Reverse Primer = 5′-GTCTCGTGGGCTCGGAGATGTGTATAAGAGACAGGACTACHVGGGTATCTAATCC-3′. These locus-specific primers were synthesized with overhanging Illumina adapter sequences. A second PCR was performed to incorporate multiplexing indices and Illumina sequencing adapters. Amplicon libraries were then purified using 0.8x AMPure XP beads (Beckman Coulter) and size verified on a Bioanalyzer 2100 (Agilent Technologies) using an Agilent High Sensitivity DNA Kit. Libraries were quantified using the Qubit™ dsDNA HS Assay Kit (Thermo Fisher Scientific), normalized, pooled, and paired-end sequenced using the MiSeq Illumina Platform.

### 2.4 Sequence analysis

Raw paired sequences were uploaded as FASTQ files and analysed using the MG-RAST server v 4.0.3 [5], [6], [7]. Reads obtained after quality control were subjected to taxonomic analysis by comparing with different ribosomal RNA databases using the open and closed-reference Operational Taxonomic Unit (OTU) picking strategy. The OTUs were classified using the Greengene 13_8 16S reference database [8]. Taxonomy assignments were made to each OTU using the RDP classifier [9] and Silvangs [10]. Finally, the sequence coverage by rarefaction analysis and the alpha diversity of species in each varillal was produced by the MG-RAST pipeline. The microbial metabolic pathways were determined based on the 16S rDNA gene data using Parallel-Meta3 software v 3.5.3 [11,12]

## Appendix Supplementary materials

Image, application 1
