# Supplementary material for: Metagenomic 16S rDNA amplicon data on bacterial diversity profiling and its predicted metabolic functions of varillales in Allpahuayo-Mishana National Reserve
Source: Data Brief. 2020 Apr 28;30:105625. doi: 10.1016/j.dib.2020.105625 (PMC7201190; doi:10.1016/j.dib.2020.105625)

**Supplementary material**

Fig. S1. Map showing the location of the Allpahuayo-Mishana national reserve in Peru


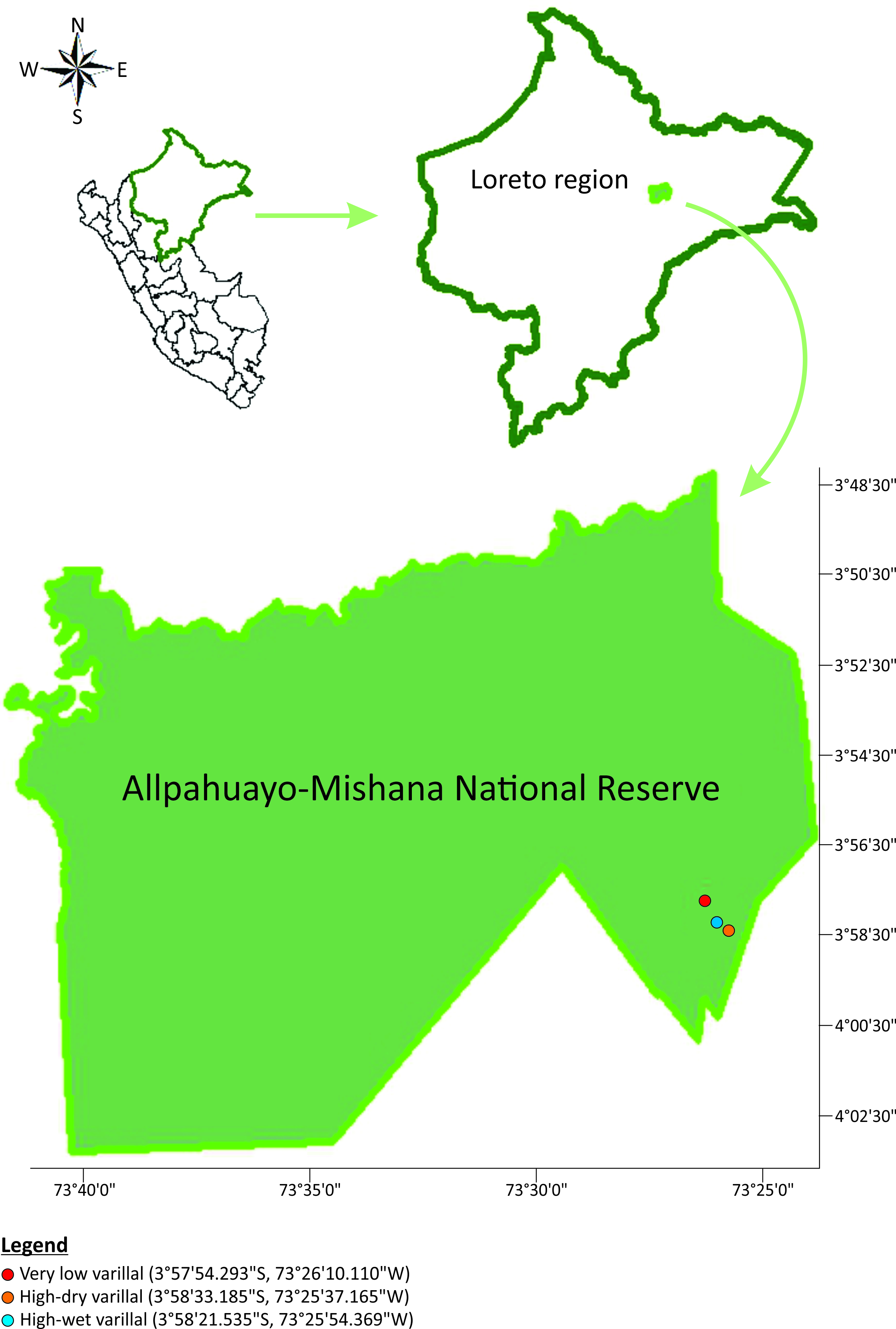


Fig. S2. Soil sample collection strategy


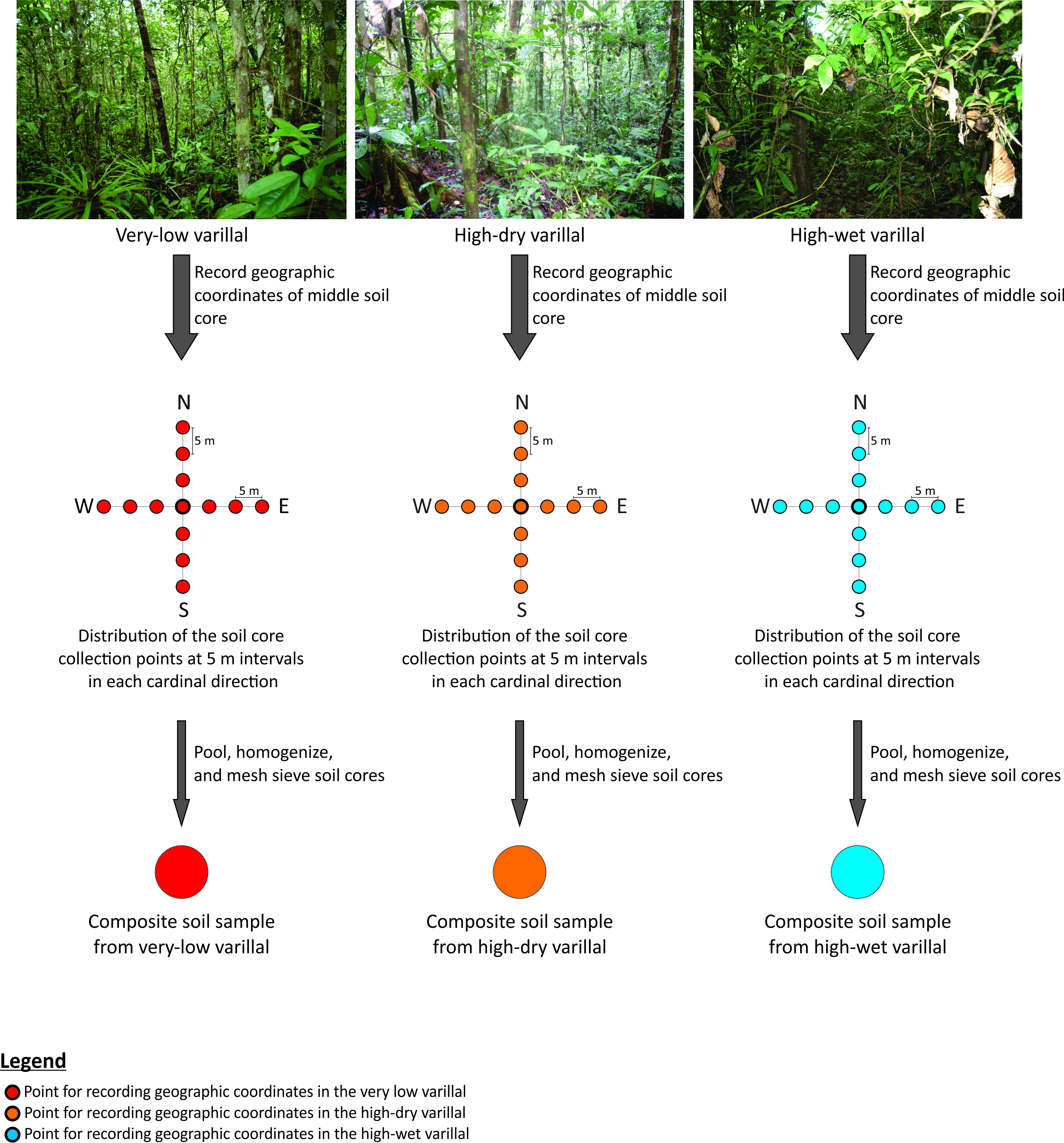

Supplement: Supplementary file 1 [file mmc1.docx]
